# The influence of charging and discharging on the thermal properties of a carbon nanotube/polyaniline nanocomposite electrode†

**DOI:** 10.1039/c9ra00151d

**Published:** 2019-03-06

**Authors:** Zheng Duan, Yufeng Luo, Zhiling Luo, Wei Yu, Changhong Liu, Shoushan Fan

**Affiliations:** Tsinghua-Foxconn Nanotechnology Research Center, Department of Physics, Tsinghua University Beijing 100084 China chliu@mail.tsinghua.edu.cn

## Abstract

In recent years, carbon nanotube/polyaniline (CNT/PANI) nanocomposites have aroused much interest because of their broad application prospects as electrodes in supercapacitors and batteries. However, a great deal of heat can be generated during fast charging and discharging processes and this can influence the efficiency of devices. In this paper, we measured the thermal properties of CNT/PANI in different oxidation states. The results indicate that within an electric potential range from −0.4 V to +0.4 V, both the thermal diffusivity and the thermal conductivity decrease obviously with potential due to the successive loss of electrons from PANI. Losing protons at higher voltages leads to a reduction in thermal conductivity but a jump in thermal diffusivity. The composite material provides an example for studying the influence of the loss or gain of electrons and protons on the thermal properties of a polymer. It also provides a superb system for thermal management through electric potential.

## Abstract 2

Fast charging/discharging process of the supercapacitors would generate a great deal of heat and influence their efficiency. In this paper, we investigate the influence of charging–discharging on the thermal properties of the CNT/PANI electrodes.

## Graphical abstract

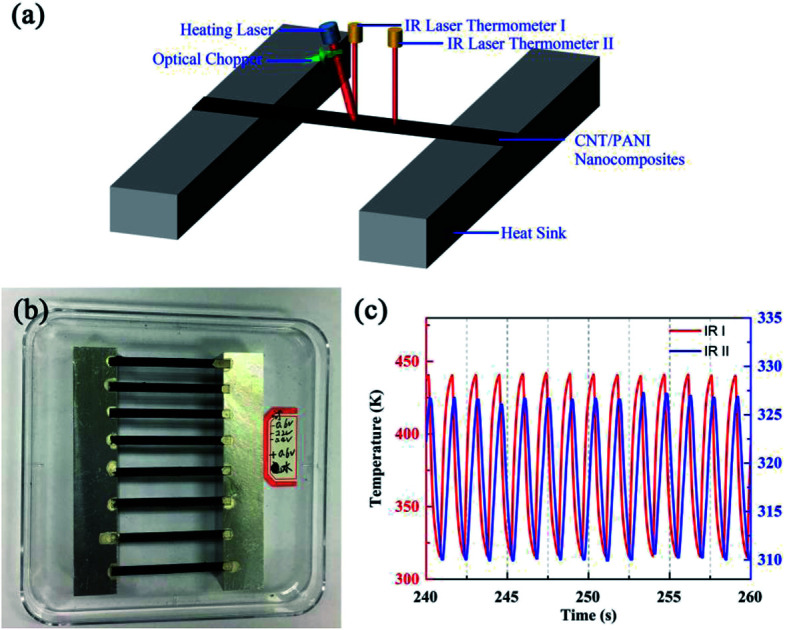

## Introduction

Polyaniline (PANI) is a polymer with remarkable electrical and electrochromic properties,^1–4^ and it is widely used in many fields, such as in sensors and anticorrosive paint.^5–7^ Carbon nanotubes (CNTs) are a nanoscale material with outstanding mechanical, electrical and thermal properties.^8–10^ CNT/PANI nanocomposites combine the virtues of both materials together and have been extensively studied.^11,12^ It has been reported that supercapacitors based on CNT/PANI nanocomposites show a specific capacitance several times larger than current high-level commercial supercapacitors.^13^ The composites can also serve as electrodes in hybrid batteries, showing both high specific power and high specific energy and excellent rechargeable characteristics.^14^ During the fast charging and discharging processes, considerable amounts of heat can be generated and this may influence the properties of devices. Therefore, it is meaningful to study the variations in the thermal properties, such as the thermal diffusivities and the thermal conductivities of CNT/PANI nanocomposites, in different oxidation states. Although the thermal properties of the two materials have been respectively studied, the influence of oxidation states on the thermal properties of such nanocomposites has not been reported previously.

Many methods have been put forward to measure the thermal diffusivity of materials. The periodic heat flux method is based on a theory originally stated by Ångström.^15^ The method has the characteristics of short test times and simple calculations, and it is widely used for thermal diffusivity measurements. Many measurement methods have been developed based on lasers, such as mirage-effect measurements,^16^ transient thermal gratings,^17,18^ and the flash method.^19^ The merits of these methods are that they are non-contact, suitable for small samples, and so on. The transient hot-strip method is appropriate for many kinds of materials and can obtain thermal diffusivity data over a wide temperature range.^20^

In this paper, we utilized the periodic heat flux method and steady-state heat method to obtain the thermal diffusivity and thermal conductivity of CNT/PANI nanocomposites, respectively. The results show that the oxidation process (within a potential range from −0.4 V to +0.4 V) will decrease both the thermal diffusivity and thermal conductivity of the nanocomposite. However, at a potential of +0.6 V, the thermal conductivity decreases while the thermal diffusivity jumps, due to a loss of protons from PANI.

## Experimental

CNT/PANI nanocomposite strips were fabricated as follows. A super-aligned CNT array was grown on a silicon substrate using the method of low-pressure chemical vapor deposition.^8,21^ The diameters of the individual MWCNTs are about 10–20 nm.^22^ The CNTs were detached from the substrate, and a CNT suspension was obtained *via* ultrasound treatment in ethanol for 20 minutes. With the help of a vacuum, the suspension was filtered through a microporous membrane, which led to a dense CNT sheet made of randomly entangled individual CNTs and CNT bundles. The membrane with CNTs adhered was dried in an oven at 330 K for 6 hours. Then the freestanding CNT sheet was peeled off the membrane. The CNT network was fully immersed in 20 ml of precooled aniline solution, whose solute concentrations were 1 M HCl and 0.05 M aniline. Then, precooled 0.05 M ammonium persulfate was slowly added to the aniline solution drop by drop. In order to allow a complete reaction, the mixture was kept at 273 K for 12 hours. Finally, the CNT/PANI sheet was washed with deionized water, and dried in an oven at 330 K for 12 hours. The thickness of the CNT/PANI sheet was about 90 μm and was quite uniform. The CNT/PANI sheet was cut into 40 mm × 3 mm strips using a high-power infrared laser. During the process of applying bias voltages, a platinum wire served as the counter electrode. Each CNT/PANI strip was subject to bias in 0.18 M sulfuric acid at a certain voltage (in the range of −0.6 V to 0.6 V in this study) for 3 minutes. Afterwards, the samples were immersed in deionized water for 30 seconds to remove residual sulfuric acid. Then the strips were dried in air for 24 hours.

## Results and discussion

The method of thermal diffusivity measurement we utilized in this study is based on one-dimensional periodic heat transfer.^15,23–25^ The heat flux can be regarded as quasi-1D diffusion in the in-plane direction, due to the large length to width ratio. The one-dimensional Fourier equation is1
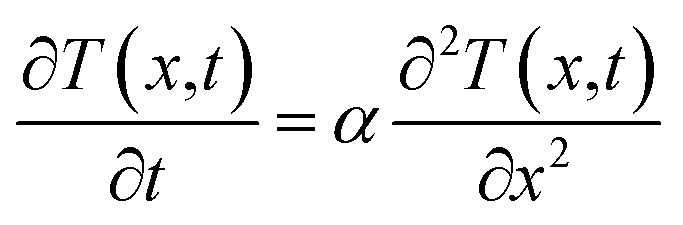
where *α* is the thermal diffusivity and *x* is the coordinate. If the boundary condition is a sinusoidal heating flux, *A* sin(*ωt*), the solution to the equation is2
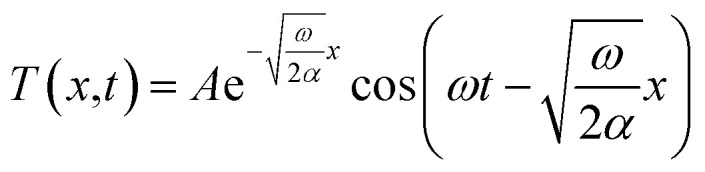
where *ω* is the angular frequency. The exponent term indicates that the amplitudes decrease as the distances increase, while the cosine term indicates that the temperature oscillates periodically with time and distance. If we measure the temperatures of two points on the sample and the distance between them is Δ*x*, we will obtain two waves with different amplitudes and time phases. The time phase difference between the two waves, Δ*t*, could be determined *via* a peak finding method. We can obtain the thermal diffusivity of the sample using the equation *α* = Δ*x*^2^/2*ω*(Δ*t*)^2^, which can be derived from eqn (2).^15^ As we have described above, all we need to determine is the distance between two points and the time phase difference between two heat waves. Although the exponential part of the equation could also be used to derive the thermal diffusivity in principle, it is much more difficult to do this than to use the time difference part in practice. This is because the heat not only conducts from the heated point to the heat sink, but it is also dissipated *via* aerial convection and radiation.

**Fig. 1:**
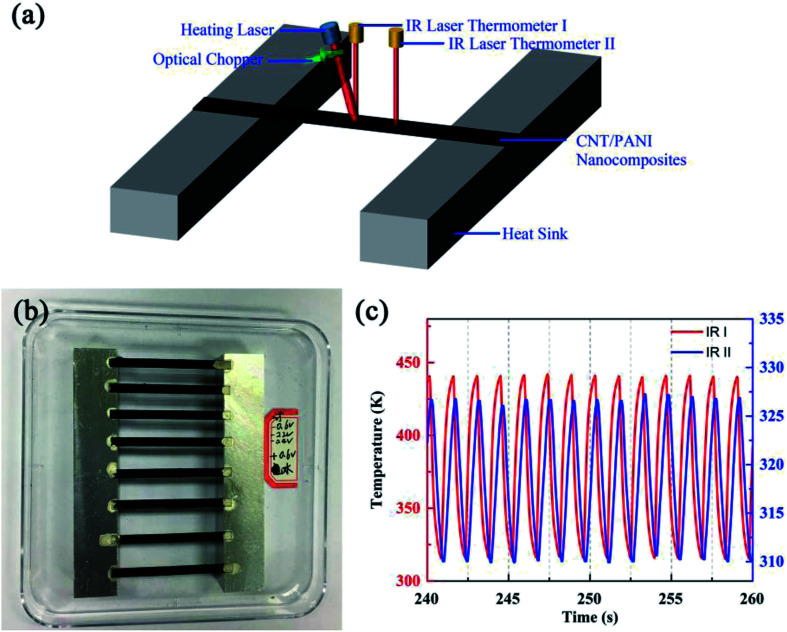
(a) A schematic diagram of the system used for thermal diffusivity measurements. An infrared laser with an optical chopper heated the center of the CNT/PANI nanocomposite strip. Two infrared laser thermometers were employed to monitor the temperatures at the center and a certain other point, respectively. A pair of aluminum blocks were used as a heat sink. (b) An optical image of the samples. The samples were adhered to the heat sink using silver adhesive. (c) The temperature distributions at the center point and the certain point on the CNT/PANI nanocomposite strips. This is one of the real-time data samples from our measurements, and obvious time phase differences and amplitudes could be observed.

Fig. 1(a) shows a schematic diagram of the system used for the thermal diffusivity measurements. Strips under bias at different potentials were pasted onto a pair of aluminum blocks with silver adhesive used to obtain perfect thermal contact. The gap between the two aluminum blocks was 34.81 mm. A near-infrared laser with a central wavelength of 980 nm was used to heat the center of the strip. The heating power of the laser was set to about 1.06 W. A convex lens was used to focus the laser totally on the sample. An optical chopper was used to generate a periodic heat source, whose frequency was controlled by a voltage source, and the voltage remained constant during all of our experiments. The heating period was about 1.45 seconds. Two Optris LS infrared thermometers were utilized to monitor the temperatures of the samples; their temperature resolution, spatial resolution and time resolution were 0.1 K, 1 mm and 1 ms, respectively. One of the thermometers monitored the central point of the strip, which exactly coincides with the heating point. The other thermometer monitored a point 5.88 mm away from the heating point. Fig. 1(b) shows an optical photograph of the samples. An extra strip (the top one) was adhered to investigate the best conditions for experiments. The two aluminum blocks were adhered to a substrate in order to allow the samples to be moved as a unit. During the experiments, the heating laser, the optical chopper and the two infrared thermometers were kept stationary. The samples were tested in turn by moving the substrate.

In order to decrease any influence from fluctuations in the surrounding environment, after the system reached the steady state, thirteen or fourteen contiguous cycles were observed and the time scale was about 20 seconds. Fig. 1(c) shows the typical monitored temperatures of a sample biased at −0.2 V obtained using the infrared laser thermometers I (red) and II (blue). The temperature curve monitored by thermometer I indicates that the curve did not sinusoidal vary with time rigorously. This is because when the heating laser heats the center of the strip directly, the temperature increases rapidly at first and then changes slowly. When the optical chopper is sheltering the heating laser, the temperature decreases promptly at first and then changes relatively slowly. The temperature curve monitored by thermometer II was more similar to a sinusoidal function due to the heat transfer process in the system. We stress that the heating laser modulated by the optical chopper resulted in square waves, rather than sine-waves. But this does not influence what we have discussed above. As we have analyzed, the key point for thermal diffusivity calculations is the time phase difference between the two thermal waves, and the distance between the measurement positions. The time phase difference is determined by the peaks of the waves, which are independent of the waveform.^25^ The figure shows the obvious time phase difference and the different amplitudes of the two temperature waves. The temperature monitored by infrared laser thermometer I ranged from about 316 K to 440 K, with an amplitude of about 124 K. The temperature monitored by thermometer II ranged from about 310 K to 327 K, with an amplitude of about 17 K. The final results relating to thermal diffusivity will be discussed together with those for thermal conductivity.

**Fig. 2:**
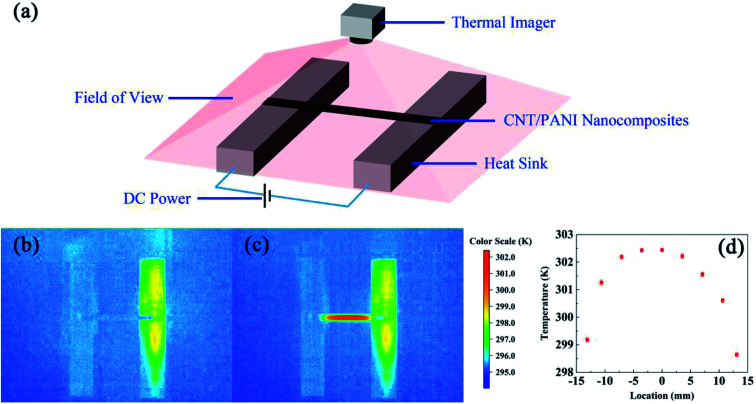
(a) A schematic diagram of the system used for thermal conductivity measurements. An infrared thermal imager was employed to monitor the temperature distribution of the strip. The sample was heated by DC power and so, differently from the thermal diffusivity measurements, only one sample could be measured at a time. (b) and (c) are two typical thermal images obtained at room temperature and under steady state heating, respectively. The apparent temperature of the right aluminum block was higher than the left one, which was induced by the reflection of light from the environment. (d) The temperature distribution of the sample. The curve approximates to a parabola, which is consistent with theoretical analysis.

The measurement method for thermal conductivity was based on a self-heating method, as shown in Fig. 2(a). The length, the width, and the thickness of the CNT/PANI nanocomposite strip were 2*L*, *w*, and *h*, respectively. The strip was adhered to a pair of aluminum blocks with silver paste to create both electrical and thermal contacts. The distance between the two aluminum blocks was about 26 mm. The strip was heated uniformly by DC power with the total power *P* = *UI*. Only one sample could be measured at each time. The temperature of the sample was monitored using an infrared thermal imager (Optris PI400), whose temperature resolution was 0.08 K. Fig. 2(b) and (c) show the system before and after turning the power on, respectively. The power supply was maintained at 0.7 V. The apparent temperature of the right aluminum block was higher than that of the left one. This was caused by the reflection of light from the environment. In order to simplify the measurements, the experiment was performed in air, rather than under vacuum. The equations and process data are derived as they would be under vacuum, and the influence of aerial convection is derived in the ESI.† The conclusion is that the obtained thermal conductivity will be slightly larger than the intrinsic thermal conductivity. According to thermal flow theory, the one-dimensional steady-state Fourier equation could be written as^26^3
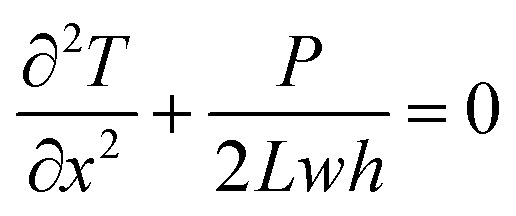
without considering aerial convection. From the equation, we could obtain the temperature distribution over the strip4
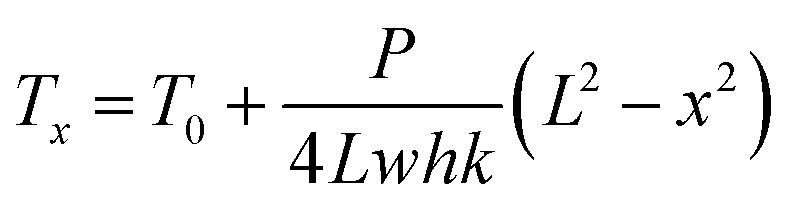
where *T*_0_ is the room temperature. The results show that the curve is a parabola, and the maximum temperature *T*_M_ is in the middle of the sample. Fig. 2(d) shows the temperature distribution over the sample, which fits with theory quite well. The slightly asymmetry at the two ends of the sample may be induced by different contact statuses. Finally, the thermal conductivity of the sample can be expressed as5
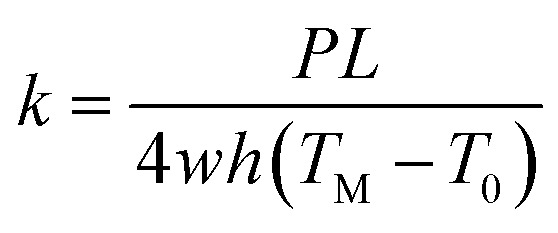
where *T*_M_ is the temperature of the middle point.

**Fig. 3:**
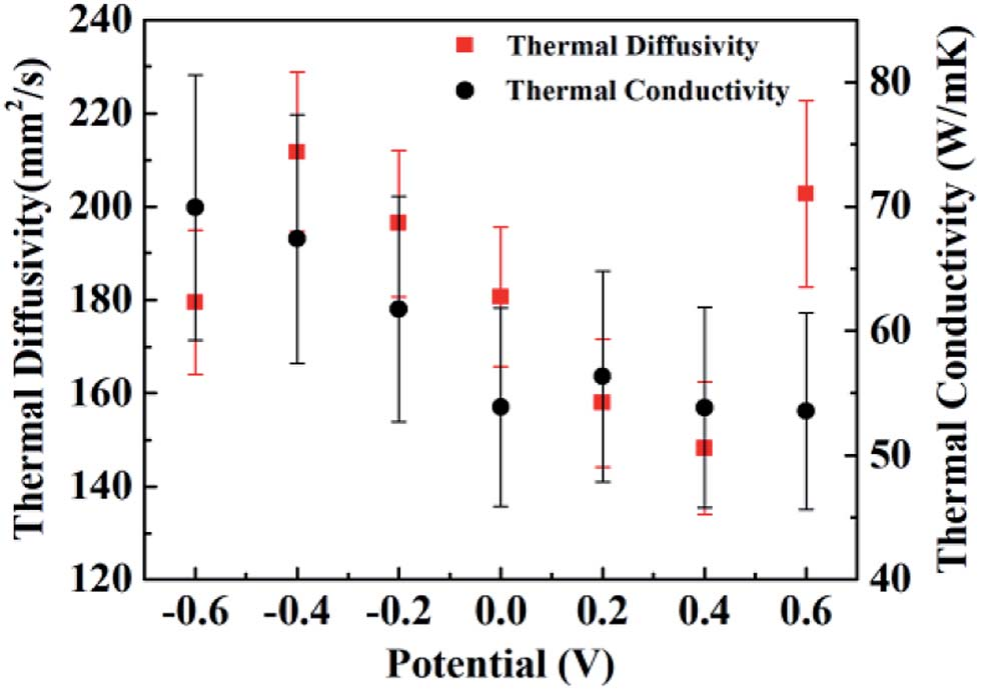
The thermal diffusivities (red squares) and thermal conductivities (black circles) of samples under bias at different potentials. The thermal diffusivities decrease over the potential range from −0.4 V to +0.4 V. A sudden drop and jump were respectively observed at −0.6 V and +0.6 V. The thermal conductivities decrease almost monotonously with potential within the range of error.

Fig. 3 shows the thermal diffusivities (red squares) and the thermal conductivities (black circles) of samples under bias at different potentials. The error bars of the thermal diffusivity data points capture the uncertainty due to fluctuations in the time differences between the two thermal waves and the sensitivities of the measurement devices. The thermal diffusivities of the CNT/PANI composites range from about 148 mm^2^ s^−1^ to 212 mm^2^ s^−1^, which are close to the values for super-aligned CNTs.^25^ The thermal diffusivities decrease almost linearly as the potential increases from −0.4 V to +0.4 V, while the thermal diffusivity of the sample under bias at −0.6 V is less than that under bias at −0.4 V, and that of the sample under bias at +0.6 V is larger than that under bias at +0.4 V. This curious phenomenon was repeatable, and another independent set of results is shown in ESI Fig. S1.† The thermal conductivities shown in Fig. 3 are mean values from the two latest independent sets of results. The error bars for the thermal conductivity data capture the uncertainty of temperature fluctuations in the steady state and the sensitivities of the measurement device, and are geometric means of the uncertainties from two independent sets of data. The thermal conductivities range from about 53.6 to 70.0 W m^−1^ K^−1^, and practically decrease as the bias potential increases within the limits of accuracy. The values of the thermal conductivities are also close to those of super-aligned CNTs.^25^ The thermal conductivities of the original two independent data sets are shown in ESI Fig. S2.† Meanwhile, the equation *k* = *C*_p_*ρα* associates the thermal conductivity *k* with the thermal diffusivity *α*, where *C*_p_ is the heat capacity and *ρ* is the density of the material. The heat capacity is discussed in the ESI.†

There have been some efforts to tune the thermal conductivities of inorganic oxides using electric potential. In a high parallel electric field, the thermal conductivity of a SrTiO_3_/KTaO_3_ system was two orders of magnitude larger than that without an electric field,^27^ and in silicon systems the thermal conductivity decreased by two orders of magnitude.^28^ At small voltages, changes in the thermal conductivity of about 11% and 30% were observed in Pb(Zr_0.3_Ti_0.7_)O_3_ and Li_*x*_CoO_2_ systems, respectively.^29,30^ As for organic compounds, only in beeswax has it been observed that the thermal conductivity is greater if an electric field is applied during solidification, and changes in the thermal conductivity have not been stated.^31^ Here, for CNT/PANI nanocomposites, over the potential range from −0.4 V to +0.4 V, the thermal conductivity and thermal diffusivity change by about 25% and 43%, respectively, which is comparable to inorganic materials. Meanwhile, it has been reported that after hundreds of charge–discharge cycles, the properties of PANI remain stable.^3,14^ Therefore, CNT/PANI nanocomposites provide a superb system for thermal management *via* an electric potential with a new mechanism and are important for further understanding the thermal properties of polymers.

**Fig. 4:**
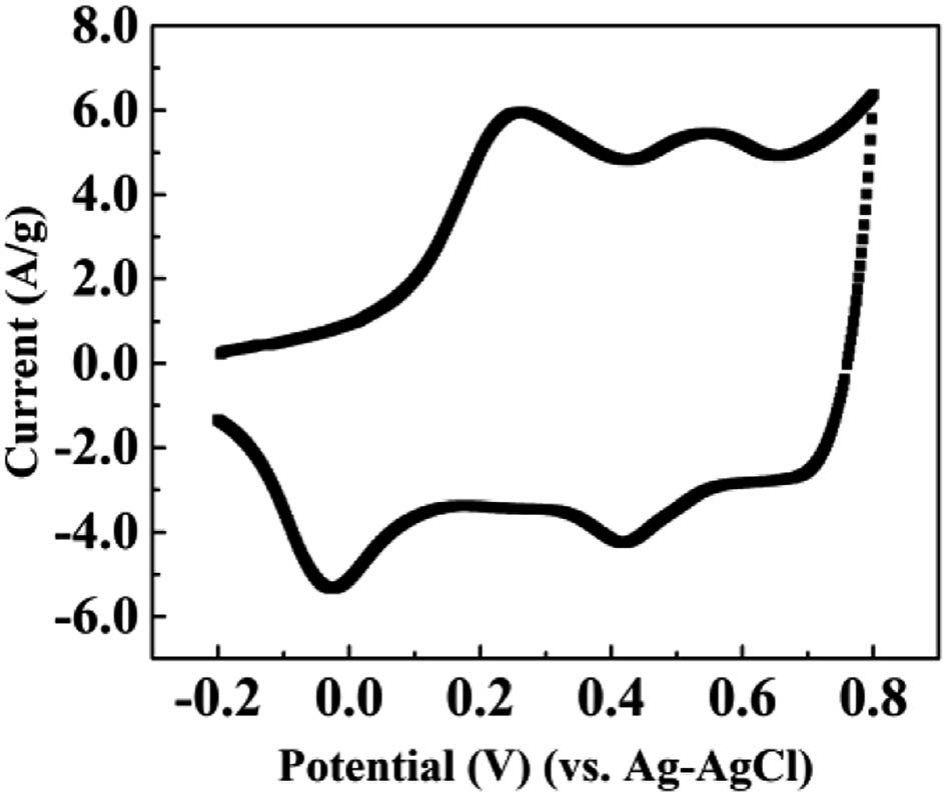
The first voltammogram scan of the CNT/PANI nanocomposite in 0.18 M H_2_SO_4_ aqueous solution with a sweep rate of 10 mV s^−1^.

The first voltammogram cycle for the CNT/PANI composite in 0.18 M aqueous sulfuric acid solution is shown in Fig. 4. The applied potential ranged from −0.2 to +0.8 V *vs.* Ag–AgCl and the scanning rate was 10 mV s^−1^. We do not demonstrate more voltammogram cycles, because the conditions for each sample are equivalent to the first cycle. There are two peaks in the voltammogram, which are at about 0.27 V and 0.54 V, respectively. The positions of the peaks are similar to those found in previous reports for pure PANI.^3,4^ This could be attributed to the idea that the CNTs were enveloped by PANI and all the chemical reactions were occurring at the PANI shell. The voltammogram demonstrated that the most suitable potential for the first reaction was 0.27 V, and it almost stopped at 0.42 V; the most suitable potential for the second reaction was about 0.54 V, and it stopped at 0.65 V. Reactions involving PANI are quite complex, because the properties depend both on the oxidation state and the protonation state.^32^ Watanabe *et al.* demonstrated that PANI could be oxidized from a leucoemeraldine base state to an emeraldine salt due to the formation of Würster-type radical cations, and it could be further oxidized to a pernigraniline salt at a higher potential.^3^ Then the pernigraniline salt could transform to a pernigraniline base. The transformation between different forms of PANI is shown in a diagram in ESI Fig. S3.† Combined with previous reports regarding the transformation of PANI,^4,32,33^ we could explain our results as follows. After the synthesis of the CNT/PANI composite, the color of the residue solution was green, which indicates that the original state of PANI wrapped on the CNTs was the emeraldine salt. When the sample was under bias at +0.2 V, the emeraldine salt began to be oxidized to the pernigraniline salt, and it was fully transformed at +0.4 V. Upon increasing the potential, such as to +0.6 V, the pernigraniline salt lost protons and became the pernigraniline base. As for the case of negative potentials, the emeraldine salt was reduced to the leucoemeraldine base, and the reduction extent increased with the potential.

We may then interpret the thermal diffusivities and thermal conductivities of the sample. Within a potential range from −0.4 V to +0.4 V, the thermal diffusivities decrease with the potential increase because of the loss of electrons. The jump in the thermal diffusivity at +0.6 V was caused by the reduction of protons. However, the reason for the sudden drop at −0.6 V is unknown according to the existing reaction diagram. It may be caused by the hydrolysis or the breaking up of PANI. The thermal conductivities almost monotonically decrease with the potential, which indicates that the losses of electrons and protons both lead to lower thermal conductivity. The specific mechanism explaining how the gain and loss of electrons influence the thermal conductivity of the polymer requires further research.

## Conclusions

In summary, we obtained the changes in the thermal diffusivity and thermal conductivity of a CNT/PANI nanocomposite under different bias potentials *via* a periodic heat flux method and steady-state method, respectively. We conclude that within the potential range of −0.4 V to +0.4 V, both the thermal conductivity and the thermal diffusivity decrease with the potential increase, and they change by about 25% and 43%, respectively. The reduction is induced by the loss of electrons. At a voltage of +0.6 V, the thermal conductivities decrease sequentially while the thermal diffusivities ramp up. The phenomenon is interpreted as arising from the loss of protons from PANI.

## Conflicts of interest

The authors declare no competing financial interest.

## Supplementary Material

RA-009-C9RA00151D-s001
