# Supplementary material for: The influence of charging and discharging on the thermal properties of a carbon nanotube/polyaniline nanocomposite electrode
Source: RSC Adv. 2019 Mar 6;9(14):7629–34. doi: 10.1039/c9ra00151d (PMC9061162; doi:10.1039/c9ra00151d)
Supplement: RA-009-C9RA00151D-s001 [file RA-009-C9RA00151D-s001.pdf]

Electronic Supplementary Material (ESI) for RSC Advances.
This journal is © The Royal Society of Chemistry 2019

**Supplymentary Information**

# The Influence of the Charging and Discharging to the Thermal Properties of Carbon Nanotube/Polyaniline Nanocomposite Electrode

Zheng Duan, Yufeng Luo, Zhiling Luo, Wei Yu, Changhong Liu* and Shoushan Fan

Tsinghua-Foxconn Nanotechnology Research Center and Department of Physics,

Tsinghua University, Beijing 100084, China

*Changhong Liu.* Email: chliu@mail.tsinghua.edu.cn

## 1. Influence of the natural air convection

Considering the air convection, the one-dimension heat transfer equation could be written as

$$\frac{d^2T}{dx^2}+\frac{UI}{2Lwhk}-\frac{H(T-T_0)}{hk}=0 \qquad (S1)$$

where $H$ is the convective heat transfer coefficient. At a certain range of temperatures, $H$ could regard as a constant. Using the boundary condition that $T(-L)=T(L)=T_0$, we could obtain the temperature distribution:

$$T=\frac{P}{4LwH}\left(1-\frac{cosh\sqrt{\frac{2H}{hk}}x}{cosh\sqrt{\frac{2H}{hk}}L}\right)+T_0 \qquad (S2)$$

Using the Taylor's expansion and substitute the coordinate of the middle point that $x$=0, we could finally get

$$k=\frac{PL}{4wh(T_M-T_0)}-\frac{HL^2}{h} \qquad (S3)$$

The result shows that the real thermal conductivity equals the thermal conductivity obtained from the vacuum minus a constant, which is determined by the natural air convection heat transfer coefficient, the length and the thickness of the sample. The air convection does not influence the behavior of the thermal conductivities we showed in Fig. 3. Usually, $H$ could be estimated as 5~15 W/m$^2$K,[1, 2] $L$ is the order of ~1 cm and $h$ is the order of 100 μm. So the second term in equation (S3) is about ~10 W/mK, which indicates that the thermal conductivity we showed in Fig. 3 is slightly larger than the real thermal conductivities.

## 2. Supporting figures

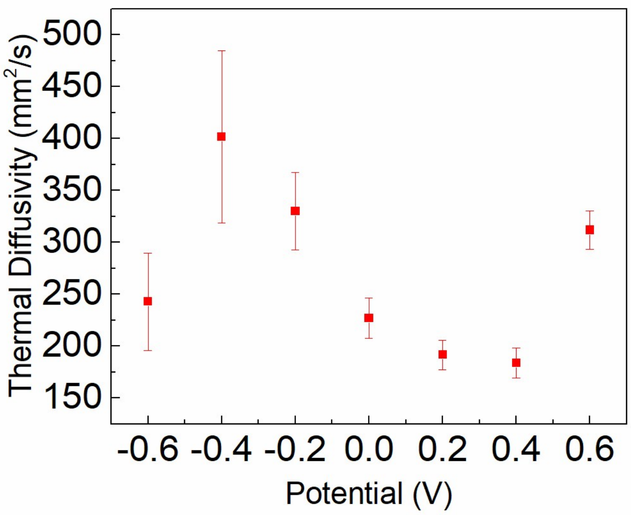


**Fig. S1** Thermal diffusivities of the sample biased at different potential.

Fig. S1 shows another independent set of thermal diffusivities. The error bars capture the fluctuations of the time difference between two thermal waves. Within the potential range from -0.4 V to +0.4 V, the thermal diffusivity decrease with potential, while jump at +0.6 V and sudden reduce at -0.6 V. This behavior is exactly same as Fig. 3.

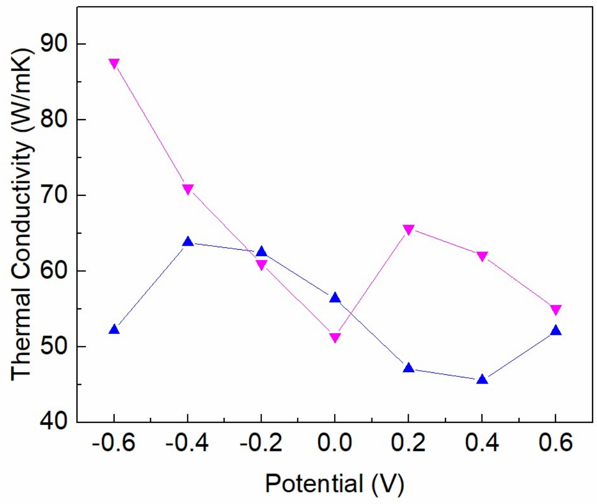


**Fig. S2** Two independent group of thermal conductivities of sample at different potential.

Fig. S2 shows the original thermal conductivities in Fig. 3. The blue curve shows a behavior extremely similar to thermal diffusivity, but it is not repeatable. The red curve first decrease within the potential range from -0.6 V to 0 V, while it jumps at +0.2 V. Then it monotonically decreases from +0.2 V to +0.6 V. As a whole, both curves decrease with the potential.

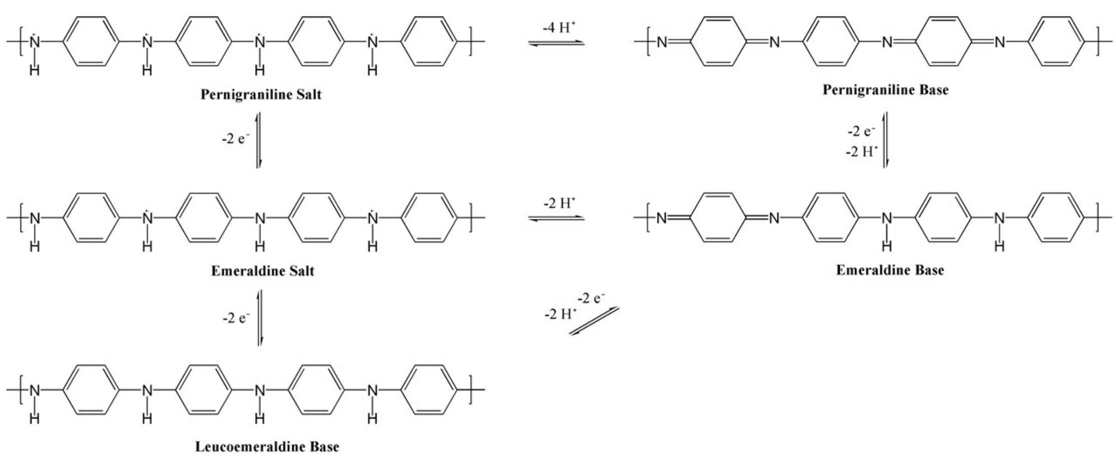


**Fig. S3** PANI transform diagram.[3, 4]

In this paper, the original state of the PANI in the CNT/PANI composite was the emeraldine salt, which was reduced to leucoemeraldine base by the negative bias, or transformed via pernigraniline salt to pernigraniline base by the positive bias.

### 3. Discussion about the heat capacity

The equation, $k=C_p\rho\alpha$, associates the thermal conductivity $k$ with thermal diffusivity $\alpha$, where $C_p$ is the heat capacity and $\rho$ is the density of the material. Though the density could be measured, we could not obtain the heat capacity accurately. First, the two physical quantities are both sensitive to the temperature, but in our experiment they were measured at different temperatures. Second, the measurement of thermal

conductivity was not perfect. So it is not reasonable to calculate the heat capacity quantitively. But it is still meaningful to analysis the heat capacity qualitatively. Within the potential range from -0.4 V to +0.4 V, both the thermal conductivities and the thermal diffusivities decrease with the potential, and the CNT/PANI composites constantly lose electrons, whose mass could be ignored. So the heat capacity remains a constant during the range. At the potential +0.6 V, the thermal conductivity reduced slightly, and the thermal diffusivity raised significantly. The density of the PANI decrease about 1% due to the loss of proton, so considering the mass of the CNTs, the overall density reduction will be less than 1%. The reduction of thermal conductivity and density could not compensate the increase of thermal diffusivity. As a result, the heat capacity of the CNT/PANI composites suddenly drop at +0.6 V. As for the potential of -0.6 V, we would conclude that the heat capacity jumped. However, as we have mentioned before, the reason is not clear and requires further study.

## Reference


1. E. M. Sparrow and P. S. Mendes, *Int. J. Heat Mass Transfer*, 1982, **25**, 293-297.
2. H.-T. Chen and J.-C. Chou, *Int. J. Heat Mass Transfer*, 2006, **49**, 3034-3044.
3. M. Jayakannan, S. Annu and S. Ramalekshmi, *Journal of Polymer Science Part B: Polymer Physics*, 2005, **43**, 1321-1331.
4. P. R. Somani and S. Radhakrishnan, *Mater. Chem. Phys.*, 2003, **77**, 117-133.
